# Impact of SARS-CoV-2 Pandemic and Lockdown on the HRSV Circulation: Experience of Three Spoke Hospitals in Northern Italy

**DOI:** 10.3390/v16020230

**Published:** 2024-02-01

**Authors:** Francesca Parola, Adalberto Brach del Prever, Virginia Deut, Giulia Costagliola, Carla Guidi, Neftj Ragusa, Antonella Tuscano, Fabio Timeus, Massimo Berger

**Affiliations:** 1 Pediatric and Neonatology Department, Ciriè Hospital, 10073 Ciriè, TO, Italy; 2 Pediatric and Neonatology Department, Ivrea Hospital, 10015 Ivrea, TO, Italymberger@aslto4.piemonte.it (M.B.); 3 Pediatric and Neonatology Department, Chivasso Hospital, 10034 Chivasso, TO, Italy

**Keywords:** Respiratory Syncytial Virus (HRSV), SARS-CoV-2 Pandemic, epidemiology, seasonality trend, HRSV prevalence

## Abstract

The SARS-CoV-2 Pandemic affected the global epidemiology of respiratory infections, including Human Respiratory Syncytial Virus (HRSV), thanks to state governments’ implementation of mitigation strategies, like the promotion of face masks and lockdowns. However, after the Pandemic, the dramatic resurge of these diseases was reported worldwide. Our retrospective study, involving three Spoke Pediatric Departments, includes all the infants under one year of age hospitalized for HRSV bronchiolitis in a period before the Pandemic period (2017–2020), during the SARS-CoV-2 Pandemic (2020–2021), and after the Pandemic (2021–2023). The primary aim was to analyze the temporal trend of HRSV in these three periods. Then, the clinical and epidemiological characteristics were analyzed to highlight the clinical differences in the affected patients, in the severity of the infections, and in the short-term outcomes. Ultimately, we analyzed the HRSV prevalence in the global bronchiolitis hospitalization over the reported periods. Overall, we included 237 patients. Before the Pandemic, the peak was recorded in January and February, while after the Pandemic, the peak was in November and December. A higher prevalence of HRSV was demonstrated after the Pandemic compared to the period before the Pandemic; overall, no difference in severity was reported. In conclusion, an increase in HRSV cases after the Pandemic has been demonstrated with an anticipated peak, while no differences were recorded in severity.

## 1. Introduction

Viral bronchiolitis is an acute respiratory illness that is the leading cause of hospitalization in young children; it represents the most common cause of acute respiratory failure in infants under one year of age in developing countries [1].

Many guidelines and consensus have been released to standardize the approach for viral bronchiolitis; the main point of discussion is regarding the definition, diagnosis, and treatment of bronchiolitis. Bronchiolitis has been diagnosed in children under one year of age, but the American guidelines use the diagnosis of bronchiolitis up to two years of age.

The diagnostic criteria of bronchiolitis, defined by an Italian Consensus referred to children under 12 months of age and included the following: an onset with rhinorrhea and/or upper respiratory tract infections; an episode of respiratory distress associated with crackles and/or wheezing, the use of accessory muscles or lower chest wall retractions, low O_2_ saturation levels, high respiratory rate relative to age, skin color changes, nasal flaring, fever, and presentation during epidemic season [2].

Human Respiratory Syncytial Virus (HRSV) accounts for 60–80% of bronchiolitis presentations.

It has been estimated that HRSV infects more than 60% of all children during the first year of life and that HRSV infects nearly all children by the time they are two years old [3,4]. HRSV is the second cause of death worldwide after malaria and the first cause of death for respiratory illness [5,6].

The clinical presentation of HRSV infections is highly variable. It may be limited to upper respiratory tract symptoms, such as fever, rhinorrhea, and congestion; the severe presentation includes bronchiolitis and pneumonia. Bronchiolitis is generally self-limiting but can lead to hospitalization due to severe respiratory distress, acute respiratory failure, or difficulty in feeding. The main risk factors for severe disease are bronchopulmonary dysplasia, age younger than 12 months, a personal history of prematurity, male sex, immunodeficiency, formula feeding, and congenital heart disease.

The primary preventive measure used is prophylaxis with Palivizumab, a humanized monoclonal antibody against HRSV; Pavilizumab is used to defend against the severe manifestations of respiratory infections due to HRSV in high-risk patients, such as children born preterm or with bronchopulmonary dysplasia [7].

HRSV is characterized by a variable epidemiology, depending on geographic area. In Italy, the HRSV circulates from mild November until the end of March. It peaks in January/February; the total circulation duration is about four months [8].

Nevertheless, HRSV circulation in the past has been influenced by previous Pandemics; for instance, in 2009, influenza H1N1 delayed the HRSV peak. This variability of seasonality could be explained by the possible viral interference and the impact of preventive measures [9].

Concerning viral interference, a virus infection could be influenced by coinfection. Recently, during the Pandemic, the virus–virus interaction has been well defined; this interaction is influenced by the virus type, the infection timing, and the interplay between the response of the host to each virus interplay. A positive interaction is reported when co-infection results in an increased disease severity and pathogenesis, while a negative interaction is reported when an infection reduces or prevents the infection and replication of a second virus (e.g., influenza A virus and HRSV) [10].

During the SARS-CoV-2 Pandemic, various restrictive measures were adopted worldwide, like the imposition of social distancing measures, the closing of schools and commercial activities, strict hygiene behaviors, the use of face masks, and travel limitations. The massive effort to contain the spread of SARS-CoV-2 also affected the circulation of other respiratory pathogens, like influenza and HRSV, with a similar transmission route (contact, droplets, and aerosol transmission) [11].

Especially during the 2020–2021 season, a few cases of bronchiolitis were reported worldwide, leading some authors to speak about “a nearly absent disease” [12]. In the 2021–2022 season, a dramatic rebound of bronchiolitis was reported in the Northern and Southern hemispheres, due to HRSV infections [12]. This data in Italy was detected by a dedicated Surveillance Network system (RespivirNet), with a weekly report of respiratory viruses’ circulation region-by-region [8].

The first report of decreased bronchiolitis was in Australia and New Zealand, where the containment of SARS-CoV-2 was excellent and quickly started to relax the SARS-CoV-2 preventive measures.

After, an unexpected unseasonal peak of bronchiolitis compared to that in the pre-Pandemic periods has been registered worldwide [13,14,15]. These data were confirmed by intercontinental reports; it shows an anticipation of the peak of bronchiolitis, due to HRSV. Also, in Italy, these data have been confirmed [16,17,18,19].

Our study aims to provide insights into the impact of the SARS-CoV-2 Pandemic on the epidemiology of HRSV infections in Spoke hospitals of our health district (ASLTO4). The secondary aim was to evaluate the differences in the clinical features of patients affected by HRSV before and after the SARS-CoV-2 Pandemic and the global increase in bronchiolitis due to HRSV.

## 2. Materials and Methods

### 2.1. Study Design

A retrospective study was carried out in Spoke hospitals in the area nearing Turin, Piedmont Region, in the northwest of Italy. Italy is divided into health districts with a hub and spoke model for referral of patients. Our local health district (ASLTO4) includes 174 cities; the overall area is characterized by a great geographical variability, from high Alpine Mountains to urban areas. The total population of this area is 504,467 people, with 3015 inhabitants under one year of age in 2022 [20]. The local healthcare system ASLTO4 is organized into five districts, with great heterogeneity in demography, population density, and infrastructures. The General Emergency Department and Pediatric Unit are present in three Spoke hospitals of Ciriè, Chivasso, and Ivrea.

We included patients under one year of age referred to our Pediatric Departments for acute bronchiolitis due to HRSV that required hospitalization over different seasons. The test used to detect the HRSV was a rapid antigen test for the qualitative detection in nasopharyngeal swabs; the test used in our hospitals is the same since 2012 (BinaxNOW HRSV CARD—Abbot).

We divided the included patients into two groups: Group A hospitalized before the SARS-CoV-2 Pandemic (from 1 September 2017 to 31 March 2018; from 1 September 2018 to 31 March 2019; and from 1 September 2019 to 31 March 2020) and Group B hospitalized after the period of the Pandemic (from 1 September 2021 to 31 March 2022 and from 1 September 2022 to 31 March 2023) when the restrictive measures were relaxed.

In order to estimate the prevalence of HRSV, we reported the total number of cases of bronchiolitis hospitalized during the same periods and the few cases of bronchiolitis in the Pandemic period (from 1 September 2020 to 31 March 2021).

The period of data collection from September to March was established based on the HRSV epidemiology in Italy [8].

We collected data about demographic variables (sex, age, months of admission). Clinical and epidemiological data were recorded (age at onset, gestational age and birth weight, weight at admission, feeding, fever) as well as a personal history of chronic illnesses such as cardiopathy, neurological disease, and bronchopulmonary dysplasia. Relevant clinical variables were documented like laboratory (C-reactive protein in a performed blood sample and Pco2 in the Blood Gas Analysis) and microbiology (coinfection by other microbiological agents). C-reactive protein (CRP) was considered normal below 10 mg/dL. The coinfection was investigated based on clinical suspicion: we usually assessed antibodies for Epstein–Barr Virus or Mycoplasma pneumonia in blood samples. In Group B, all patients were tested for SARS-CoV-2 with an antigen rapid test on a nasopharyngeal swab for hospital admission. We reported radiograph results and short-term outcomes (length of hospital stay, complications, Hub hospital transfer). Treatment during hospitalization (low- or high-flow oxygen supplementation, nebulized therapy, steroids, antibiotics, and intravenous hydration) and discharged therapy data were recorded in all patients.

Firstly, the temporal trend of HRSV bronchiolitis after the SARS-COV-2 Pandemic (Group B) was compared to that of the previous period (Group A).

Then, the clinical and epidemiological characteristics were compared to assess if there were differences in the patients affected, in the severity of the infections, and the short-term outcomes.

Finally, we analyzed the HRSV prevalence in global bronchiolitis hospitalization in the same periods, both over-reported and during the Pandemic.

The study was conducted with full conformance to the principles of the Declaration of Helsinki. In accordance with the current legislation, this research is not among the types that requires a formal permission from an ethics committee. This is a secondary use of data for research purposes for which specific informed consent was requested ab initio from patients who would undertake a treatment process.

### 2.2. Statistical Analysis

Univariate analysis was performed with the Chi-square or Fisher’s test for dichotomous variables, while the Kruskal–Wallis test for nonparametric measures was used for continuous variables [21]. The Kaplan–Meier statistics were used to define the probability of success [22]. The difference between groups was calculated with a log-rank test [23].

The univariate analysis was conducted using Vassar Stats (Statistical Computation Web Site), while the Kaplan–Meier statistics were performed using the NCSS software for Windows (https://www.ncss.com/; accessed on 4 December 2023). A *p*-value below 0.05 was defined as statistically significant.

## 3. Results

Overall, we hospitalized 468 bronchiolitis patients, 272 before the SARS-CoV-2 Pandemic (from 1 September 2017 to 31 March 2018; from 1 September 2018 to 31 March 2019; and from 1 September 2019 to 31 March 2020), 3 during the Pandemic (from 1 September 2020 to 31 March 2021), and 193 after the period of the Pandemic (from 1 September 2021 to 31 March 2022 and from 1 September 2022 to 31 March 2023).

In 237 patients, the HRSV was detected on the nasal swab, and for this reason, they were enrolled in the study: 109 in the Group pre-pandemic (named Group A) and 128 in the Group post-pandemic (named Group B). No HRSV bronchiolitis patient was admitted during the SARS-CoV-2 pandemic.

### 3.1. Seasonality

**Figure 1:**
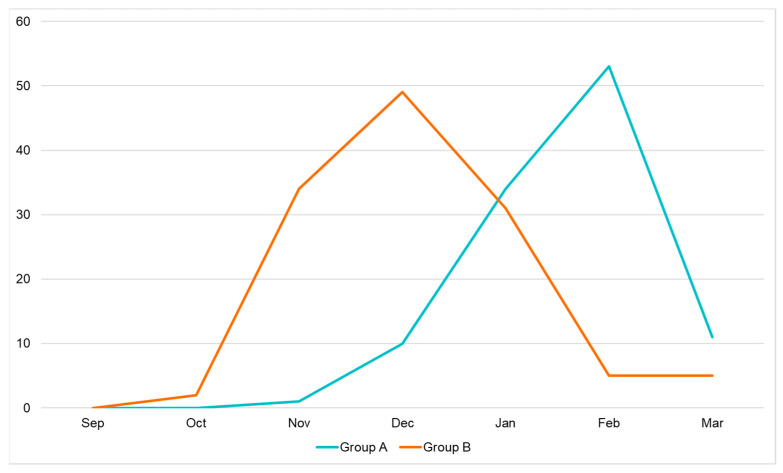
Seasonal trend of HRSV bronchiolitis.

Hospitalization for HRSV bronchiolitis followed a distinct seasonal trend in the two groups, as shown in Figure 1.

In Group A (patients admitted in the period before the SARS-CoV-2 pandemic), the peaks in admissions occurred between November and March, usually lasting 2–4 months. Cases increased significantly in December and peaked in January and February, with only a few cases reported in March.

On the other hand, in Group B (patients admitted after the SARS-CoV-2 pandemic), an anticipated peak was reported. Indeed, bronchiolitis started slowly in October, peaked in November and December, and slowly decreased during February and March.

The distribution of hospitalization month-by-month is summarized in Table 1.

### 3.2. Clinical and Epidemiological Characteristics of Hospitalized Patients

The demography and clinical characteristics of patients hospitalized in all periods that were considered (pre-pandemic, during pandemic, and after pandemic) are provided in Table 2.

In Group A, males were more represented than females (51% vs. 49%); gestational age was reported in 98% of patients. Preterm infants accounted for about 11%; half of the patients had a gestational age between 30 and 34 weeks, and the remaining patients had a gestational age between 35 and 36 weeks. In this group (patients admitted before SARS- SARS-CoV-2 pandemic), no patient had a gestational age under 30 weeks. Two patients had bronchopulmonary dysplasia, two infants had chronic heart conditions, one had a neurological disease like epilepsy, and no infant with immunodeficiency was recorded. At the time of diagnosis, most patients (48%) had an age between one and three months while 34% of them were under one month of age; furthermore, 13% had an age between four and six months and 5% of them were over six months of age. Breastfeeding was reported in 58% of all patients, while formula feeding was reported in 34% of patients of the Group A. Furthermore, 6% of infants had already been weaned. In one patient, during the hospitalization, a coinfection of Epstein–Barr Virus (EBV) was detected.

As specified before, no HRSV bronchiolitis was found during the SARS-CoV-2 pandemic in our hospitals.

In Group B (patients admitted after the SARS-CoV-2 pandemic), more males than females (61% vs. 39%) were represented, and the gestational age was reported in 95% of patients. Among the patients for whom the gestational age was available, preterm infants were calculated to be 12% of all patients; most of these patients (67%) had a gestational age between 35 and 36 weeks, while 27% of them had a gestational age between 30 and 34 weeks, and only one infant had a gestational age of 25 weeks. Two patients had bronchopulmonary dysplasia, and one infant had a neurological disease with hypotonia. No infants affected by immunodeficiency or chronic heart conditions were recorded. When hospitalized, most patients (48%) were newborns under one month of age, while 23% had an age between one and three months, and 17% were between four and six months. Overall, 11% of infants were over six months old of age. Breastfeeding was reported in 66% of all patients, while formula feeding was reported as feeding modality in 20% of all patients. Furthermore, 10% of infants had already been weaned. Concerning the coinfections, we reported three cases in whom another pathogen was found. In one case, we also found an EBV first infection; in the second patient, a Mycoplasma pneumoniae co-infection occurred, and in the last patient, the nasal swab tested positive for both HRSV and SARS-CoV-2.

In the description of the demographic features of patients involved in the study, we found a remarkable difference in the age of the infants; in Group B (post-pandemic), there were more newborns. No statistical differences in sex, gestational age, feeding, and comorbidity were recorded.

Concerning the prophylaxis with Palivizumab, only one patient in Group B was hospitalized for bronchiolitis due to HRSV; he was born at 31 weeks of gestational age and needed Continuous Positive Airway Pressure (CPAP) at birth. He also presented pneumothorax in the hours after birth. During the hospitalization for bronchiolitis, he did not need oxygen supplementation and was admitted to the hospital for three days.

Laboratory, X-ray findings, and treatment during hospitalization are provided in Table 3.

There were no significant differences between the two groups in X-ray findings, while there was a remarkable difference in CRP values.

Concerning the treatment, some differences were remarkable. Antibiotics and IV Hydration were less used in Group B; in Group A, 38% received an antibiotic therapy compared to the 19% of patients in Group B. Similarly, in Group B, only 18% of patients were treated with IV hydration (vs. 29% in Group A).

There were no statistically significant differences in the type of respiratory support and the length of respiratory support in days.

**Figure 2:**
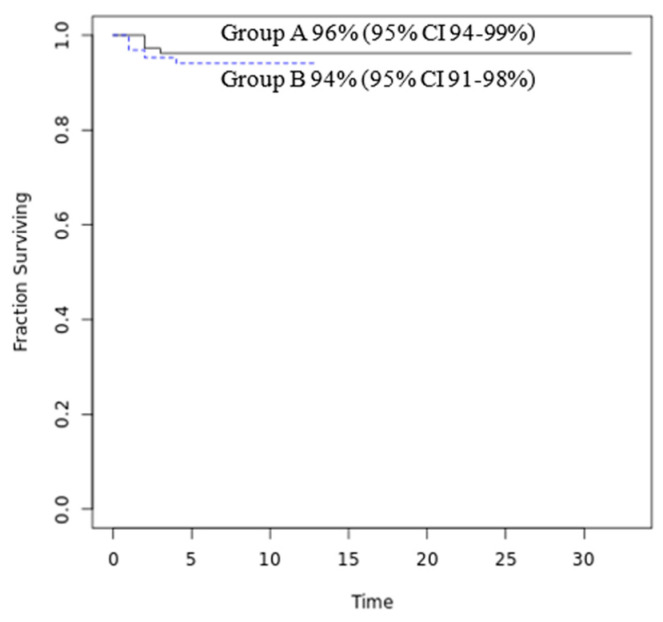
Probability of treatment success (discharge to home). Notes: gray line: Group A and light blue line: Group B.

Finally, concerning the short-term outcome, the complications, the length of hospital stay, and the need to transfer to a Hub hospital were similar in the two groups. These data are provided in Table 4. Figure 2 reports the probability of treatment success (discharge to home without transfer to a Hub hospital with PICU or NICU): 96% of patients in Group A and 94% in Group B.

### 3.3. HRSV Prevalence

In the period before the SARS-CoV-2 Pandemic (from 1 September 2017 to 31 March 2018; from 1 September 2018 to 31 March 2019; and from 1 September 2019 to 31 March 2020) in the three ASLTO4 Pediatric Departments, 272 patients were hospitalized with the diagnosis of bronchiolitis, and in 109 (40%) of them, HRSV (Group A) was detected on the nasal swab.

During the SARS-CoV-2 Pandemic (from 1 September 2020 to 31 March 2021), when restrictive measures were adopted, 3 patients were hospitalized for bronchiolitis, and HRSV was not detected.

After the SARS-CoV-2 pandemic (from 1 September 2021 to 31 March 2022 and from 1 September 2022 to 31 March 2023), the total number of hospitalizations for bronchiolitis were 193. In 128 cases (66%), the detected agent was HRSV (Group B).

Therefore, we can demonstrate a significant increase in the prevalence of HRSV after the SARS-CoV-2 Pandemic; moreover, we noticed a significant drop in the cases of bronchiolitis, but, above all, we found the complete absence of HRSV infection in the Pandemic period.

**Figure 3:**
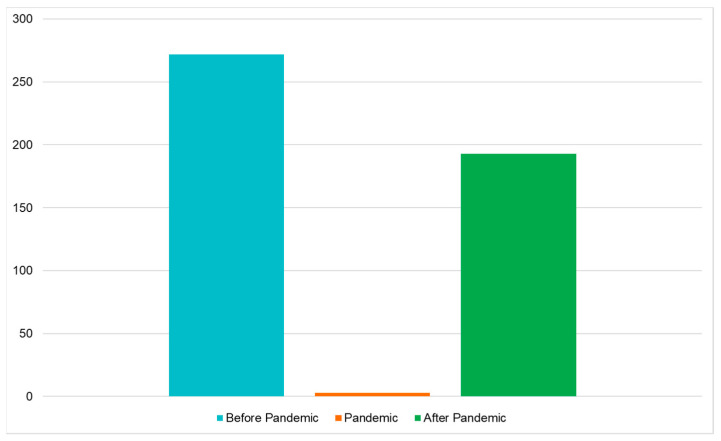
Hospitalization for bronchiolitis before, during, and after SARS-CoV-2 Pandemic.

**Figure 4:**
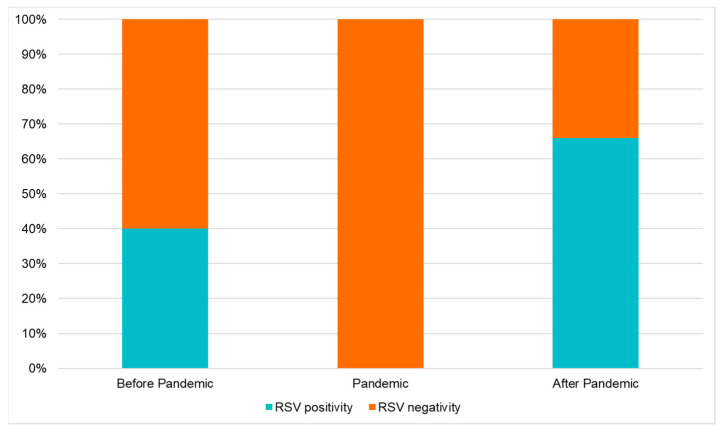
Prevalence of HRSV detected in bronchiolitis hospitalized before, during, and after the SARS-CoV-2 Pandemic.

All these data are summarized in Table 5 and Figure 3 and Figure 4.

## 4. Discussion

The Pandemic shows the importance of respiratory viruses’ circulation surveillance. Respiratory infections are very common in infants and their severity varies based on the host features (for instance, the presence of prematurity or cardio-pulmonary broncho dysplasia). Above all causes of respiratory infection, viral bronchiolitis is the most frequent lower respiratory tract infection and the leading cause of hospitalization in children less than twelve months of age. HRSV is the virus most involved in severe bronchiolitis, and its prevalence shows territorial differences.

Our study analyzes the characteristics of infants hospitalized for acute HRSV bronchiolitis in the three Pediatric Departments of ASLTO4 before, during, and after the SARS-CoV-2 Pandemic.

Our data highlight that after the SARS-CoV-2 Pandemic, the HRSV epidemic started earlier than usual. We showed a peak in November–December in Group B (post-pandemic), while in Group A (pre-pandemic), the peak was reached in January–February. This confirms new epidemiological trends of HRSV infection as reported worldwide [18,24,25,26,27]. 

The surveillance of the seasonality of HRSV is very important for the improvement and adaptation of the prevention measures. The logistics and timing are very important to optimize its prevention results; if the prophylaxis begins months before the HRSV season, the protection could wane before the end of the epidemic, leaving infants susceptible to HRSV. Similarly, if the HRSV season starts earlier than the prophylaxis, high-risk infants remain vulnerable. Considering the new epidemiological trends, in Piedmont, prophylaxis with Palivizumab in high-risk patients begins in October, starting from 2022, providing the first dose before the onset of the circulation of HRSV. This highlights the importance of improving and updating the system of local surveillance that started in Italy in 2019–2020 as the Influenza Surveillance Network (Respirvirnet) system.

In accordance with the current literature, no differences in the severity of HRSV infection have been demonstrated in our study; other authors have reported no differences in X-ray findings, short-term outcomes (like complications, the length of hospitalization, the type of respiratory support, the length of oxygen supplementation, and the need of transfer to a Hub hospital) [28,29,30]. The only remarkable difference in our study is in the results of the CRP dosage; in Group A (pre-pandemic), its average value was higher than that in Group B (post-pandemic).

Other studies, similar to this one, were conducted in Italy. It is very interesting to note that studies conducted in Sicily, a region in the South of Italy, have a similar trend of HRSV infection. The authors did not similarly report a variation in the disease severity [31,32]. Piedmont (in the northwest of Italy) and Sicily (an island in the south of Italy) have very different climate and air characteristics. The similar results in HRSV trends suggest that even in parts of Italy where there is different weather due to the different latitudes, the HRSV epidemiological trend, the severity of the disease, and its prevalence are similar. 

Interestingly, we showed remarkable differences in treatment; fewer antibiotics were used in Group B. This suggests better adherence to the guidelines, making an important effort to reduce the development of antibiotic resistance. On the other hand, a higher CRP value was found in Group A; this finding may explain the higher use of antibiotics in this group of patients: clinicians had more suspicion of bacterial complications, so they prescribed antibiotics. Lastly, by reducing the rate of IV hydration over the years, the patients were encouraged to maintain oral feeding with a higher number of fractionated meals, as suggested by the national guidelines [4]. 

Looking at the HRSV prevalence, during the SARS-CoV-2 pandemic, we observed a drop in bronchiolitis hospitalizations. We reported three patients that were hospitalized for bronchiolitis, but no case of HRSV infection was found. These data are in agreement with other studies and the epidemiological surveillance of viruses that show a global reduction in respiratory infections that share the transmission path with SARS-CoV-2, like influenza and HRSV [26,33]. Given that the transmission of HRSV occurs through droplets, the containment measures of the SARS-CoV-2 Pandemic (like the use of face masks, social distancing, smart working, and the closure of schools) have led to the reduction in HRSV transmission [11,34,35]. 

Moreover, there is a strong correlation between environmental conditions, for example, weather and air pollution, and the incidence of HRSV. A study shows a correlation between HRSV transmission, benzene levels, and humidity, while there is an inverse correlation with temperature [36]. Given that a significant reduction in air pollutants such as benzene was recorded during the Pandemic, it can be hypothesized that the pollution reduction helped to decrease the circulation of HRSV. It is very hard to quantify the contribution, but we can suggest that it is not comparable with restrictive measures. We aim to explore such hypotheses in future studies.

The prevalence of HRSV in our study group increased significantly after the SARS-CoV-2 Pandemic, confirming previously published data; we reported an HRSV surge when the prevention measures were relaxed, and the social interactions increased. This finding confirms the main role of social distance measures in the containment of HRSV circulation.

Furthermore, the “immunity debt” played a role in the increased circulation of HRSV: during the Pandemic period, the cohort of HRSV-naïve patients expanded. It happened for two reasons: (1) due to the presence of children who have never had HRSV infections and (2) due to the reduction in immunity duration, which decreased during the time and without re-exposure to HRSV. This is confirmed by studies that show an increased number of older infants affected by HRSV [36].

In our study, there is a remarkable difference in newborns hospitalized for HRSV bronchiolitis comparing pre- and post-pandemic, with 48% of newborns in Group B vs. 34% of newborns in Group A. This result is due to the reduced exposure to respiratory viruses not only in children but also in pregnant women; above all, it is known that the infection in the third trimester of pregnancy could protect newborns against HRSV infections through antibodies contained in breast milk and those transferred transplacentally [37,38,39]. 

Our study has some limitations. Our analysis is conducted in Hub hospitals with a small sample size, due to the characteristics of our health district as detailed below: First of all, a small percentage of children aged less than one year live in our district. Furthermore, the study is based on a retrospective data collection of hospital records; as a consequence, this study may be subject to information bias due to the lack of data or incomplete hospital records. In addition, we only reported data about hospitalized patients with bronchiolitis, while patients who visited the Emergency Department and were discharged or treated by general pediatric practitioners were not included. Consequently, the global prevalence and the incidence of HRSV may be underestimated.

The main strength of our study is that it was performed in three Spoke hospitals that are a part of the same health district (ASL); therefore, clinicians had the same devices for the treatment of respiratory failure and the same tests to detect HRSV. The test used was an antigen test that was less sensitive and specific than molecular methods, but the same test was used in the three hospitals and in the different analyzed periods. Furthermore, clinicians used the same protocols of treatment and had the same criteria of transfer to Hub Hospital because of the lack of Pediatric Intensive Care Unit in our health district.

## 5. Conclusions

The SARS-CoV-2 Pandemic has changed the epidemiological trend of HRSV infections in our territory. In detail, the bronchiolitis season started earlier than usual after the Pandemic; this is reported in our study in accordance with the data records from other countries. The unusual resurgence of HRSV infection was not associated with an increased severity of the illness in our study group. In addition, we reported an increase in the prevalence of HRSV bronchiolitis hospitalized after the Pandemic, with a high proportion of newborns possibly due to the “immunity debt” and the lower exposure in pregnant women.

The surveillance of the circulation of the respiratory virus is necessary to adapt the preventive measures and the hospital activity organization to the seasonal changes; indeed, the analysis of changes in seasonality allows high-risk patients to receive the optimal level of prevention with the correct prophylaxis while hospitals reorganize their activities. It may also imply transferring patients from the Hub to the Spoke hospitals, leading to a remarkable reduction in costs. Lastly, a lesson learned during the Pandemic period was that the simple preventive measures should not be forgotten, because they can markedly reduce HRSV circulation; this finding underlines the importance of the strict hygiene behaviors and the utilization of face masks by healthcare workers, who predominantly deal with high-risk patients.

## Figures and Tables

**Table 1:** Distribution of the seasonality of infections in the two groups.

	Group A N = 109	Group B N = 128	*p*-Value
September	0 (0%)	0 (0%)	<0.0001
October	0 (0%)	2 (2%)	
November	1 (1%)	36 (28%)	
December	10 (9%)	49 (38%)	
January	34 (31%)	31 (24%)	
February	53 (49%)	5 (4%)	
March	11 (10%)	5 (4%)	

**Table 2:** Demographic and clinical characteristics of patients.

		Group A N = 109	Group B N = 128	*p*-Value
Gender	Male	56 (51%)	78 (61%)	0.14
	Female	53 (49%)	50 (39%)	
Median Age (months)		2 (IQR 1–3)	2 (IQR 1–4)	0.85
Prematurity	Yes	12 (11%)	15 (12%)	0.36
	No	95 (87%)	106 (83%)	
	Unknown	2 (2%)	7 (5%)	
Months at diagnosis	<1	37 (34%)	62 (48%)	0.0012
	1–3	52 (48%)	30 (23%)	
	4–6	14 (13%)	22 (17%)	
	6–12	6 (5%)	14 (11%)	
Weight at diagnosis	<4000 g	21 (19%)	22 (17%)	0.21
		31 (28%)	34 (26%)	
	>5000 g	57 (52%)	69 54%)	
Feeding	Breast-feed	63 (58%)	84 (66%)	0.059
	Artificial	37 (34%)	26 (20%)	
	Weaned baby	7 (6%)	13 (10%)	
Comorbidity	Yes	6 (5%)	10 (8%)	0.6
	No	102 (93%)	118 (92%)	
Coinfection	Yes	1 (1%)	3 (4%)	0.62
	No	108 (99%)	125 (96%)	
Fever	Yes	45 (41%)	31 (24%)	0.0054
	No	64 (59%)	97 (76%)	
Profilaxed with Palivizumab		0 (0%)	1 (0,78%)	1

**Table 3:** Laboratory, X-ray findings, and treatment during hospitalization.

		Group A N = 109	Group B N = 128	*p*-Value
		1.98 (0–63.5)	1.19 (0–60.3)	0.039
Chest X-rays (number)	Yes	23 (21%)	19 (15%)	0.23
	No	86 (79%)	109 (85%)	
Chest X-rays (pathologic results)		13 (12%)	10 (8%)	0.27
Higher level of pCO2 (mmHg)		46 (28.2–64.1)	48 (31.7–74)	1
Low-flow Oxygen	Yes	64 (59%)	79 (62%)	0.69
	No	45 (41%)	49 (38%)	
Low-flow Oxygen days		3 (0–8)	3 (0–9)	0.76
High-flow Oxygen	Yes	29 (27%)	33 (26%)	1
	No	80 (73%)	94 (73%)	
High-flow Oxygen days		4 (1–8)	5 (1–11)	0.38
Fraction of inspired Oxygen %		35 (21–50)	34 (25–65)	0.56
Aerosolized drugs	Yes	96 (88%)	122 (95%)	0.054
	No	13 (12%)	6 (5%)	
Antibiotics	Yes	42 (38%)	24 (19%)	0.00082
	No	67 (61%)	104 (81%)	
Steroids	Yes	13 (12%)	17 (13%)	0.84
	No	96 (88%)	111 (87%)	
Hydration IV	Yes	32 (29%)	23 (18%)	0.045
	No	77 (71%)	105 (82%)	

Notes: CRP: C-reactive protein; pCO_2_: partial pressure of carbon dioxide; and IV: intravenous.

**Table 4:** Short-term outcome.

		Group A N = 109	Group B N = 128	*p*-Value
Complications	Yes	12 (11%)	12 (9%)	0.82
	No	97 (89%)	116 (91%)	
Hospitalization (days)		6 (2–33)	5 (1–13)	0.051
Transfer to a Hub hospital	Yes	4 (4%)	7 (5%)	0.55
	No	105 (96%)	121 (94%)	

**Table 5:** Cases of HRSV infections in patients hospitalized for bronchiolitis.

	2017–2020	2021–2023	*p*-Value
HRSV positive	109 (40%)	128 (66%)	<0.0001
HRSV negative	163 (60%)	65 (34%)	

## Data Availability

The collected data are available from the corresponding authors.
